# FASTING-MIMICKING DIET REDUCES RISK FACTORS FOR AGING-RELATED DISEASES IN PRECLINICAL AND CLINICAL STUDIES

**DOI:** 10.1093/geroni/igz038.962

**Published:** 2019-11-08

**Authors:** Sebastian Brandhorst

**Affiliations:** University of Southern California, Los Angeles, California, United States

## Abstract

Prolonged fasting promotes stress resistance, but its effects on longevity are poorly understood. Calorie restriction or major dietary composition changes can have profound effects on healthy aging but the inability of many subjects to adhere to chronic and extreme diets together with the potential of adverse effects limit their application. Fasting-mimicking diets (FMDs) are effective in increasing health and lifespan, possibly by inducing stem cell-based regeneration, or as therapies in mouse models of a variety of diseases. FMDs reduce cancer incidence/progression, modulate the immune response, reduce immuno-senescence, ameliorate or reverse disease progression of multiple sclerosis, Type I and Type II diabetes, and reverse inflammatory bowel disease pathology. In a randomized clinical trial, markers/risk factors for metabolic syndrome and other age-related diseases were favorably impacted after completion of 3 FMD cycles. These effects were larger in participants at risk for age-related diseases. Conclusions: We conclude that the FMD was safe and feasible in rodent and human studies. Larger studies on patients with diagnosed diseases are necessary to determine its impact on diabetes and cardiovascular disease treatment.

